# Association Between Genetic Variants in *TNF*, *IL6*, and *IL1B* Genes and Severity of COVID-19: A Cross-Sectional Study of Patients from Southern Brazil

**DOI:** 10.3390/diagnostics15111403

**Published:** 2025-05-31

**Authors:** Mariléa Furtado Feira, Renan Cesar Sbruzzi, Miriãn Ferrão Maciel-Fiuza, Vitória Carolina Griebeler, Tatiana Schaffer Gregianini, Letícia Garay Martins, Nathan Araujo Cadore, Jose Artur Bogo Chies, Thayne Woycinck Kowalski, Fernanda Sales Luiz Vianna

**Affiliations:** 1 Postgraduate Program in Genetics and Molecular Biology (PPGBM), Department of Genetics, Institute of Biosciences, Universidade Federal do Rio Grande do Sul, Porto Alegre 91501-970, RS, Brazil; mfeira@hcpa.edu.br (M.F.F.); rsbruzzi@hcpa.edu.br (R.C.S.); mirianferrao93@gmail.com (M.F.M.-F.); cadorenathan@gmail.com (N.A.C.); jabchies@terra.com.br (J.A.B.C.); tkowalski@hcpa.edu.br (T.W.K.); 2 Laboratory of Genomic Medicine, Center of Experimental Research, Hospital de Clínicas de Porto Alegre (HCPA), Porto Alegre 90035-903, RS, Brazil; vi.griebeler@gmail.com; 3 Laboratory of Immunobiology and Immunogenetics, Department of Genetics, Institute of Biosciences, Universidade Federal do Rio Grande do Sul, Porto Alegre 91501-970, RS, Brazil; 4 Laboratório Central do Centro Estadual de Vigilância em Saúde da do Rio Grande do Sul (LACEN), Secretaria da Saúde do estado do Rio Grande do Sul (LACEN/CEVS/SES-RS), Porto Alegre 90620-000, RS, Brazil; tatiana-gregianini@saude.rs.gov.br; 5 Centro Estadual de Vigilância em Saúde da Secretaria de Saúde do estado do Rio Grande do Sul (CEVS/SES-RS), Porto Alegre 90620-000, RS, Brazil; leticia-martins@saude.rs.gov.br; 6 Laboratory Genetics Unit, Medical Genetics Service, Hospital de Clínicas de Porto Alegre (HCPA), Porto Alegre 90035-903, RS, Brazil

**Keywords:** SARS-CoV-2, inflammatory response, genetic polymorphisms, disease severity, Brazilian population

## Abstract

**Background/Objectives:** Genetic variants in cytokine genes such as *IL1B*, *IL6*, and *TNF* may influence inflammatory responses to SARS-CoV-2 and affect disease severity. This study investigates the role of these variants in relation to COVID-19 outcomes, including hospitalization, ICU admission, and mortality. **Methods:** A total of 500 unvaccinated individuals from southern Brazil diagnosed with COVID-19 via RT-PCR were analyzed. DNA was extracted from nasopharyngeal swabs and genotyped for functional variants selected based on evidence of regulatory function and prior associations with inflammatory outcomes—*IL1B* (rs4848306, rs1143623, rs16944, rs1143627), *IL6* (rs1800795, rs2069832, rs2069840, rs2069845), and *TNF* (rs1799964, rs1800630, rs1799724, rs1800629, rs361525). Multivariate logistic regression analysis, adjusted for sex and age, was employed to assess the association between these genetic variants and severe clinical outcomes. **Results:** The results indicated that the *IL1B* rs16944-AG (OR: 1.98 [95% CI: 1.22–3.23], *p* = 0.006) and *TNF* rs1799964-CT (OR: 1.97 [95% CI: 1.22–3.22], *p* = 0.006) genotypes were associated with the need for hospitalization, while TNF rs1800630-AA (OR: 2.37 [95% CI: 1.08–5.33], *p* = 0.034) was associated with ICU admission. Additionally, the CC genotype of *TNF* rs1799964 was associated with a higher risk of mortality (OR: 3.73 [95% CI: 1.21–14.37], *p* = 0.034). **Conclusions:** Genetic variants—specifically *IL1B* rs16944 and rs1143627, and *TNF* rs1799964 and rs1800630—were associated with COVID-19 severity and should be further investigated in larger studies to evaluate their potential as predictive markers of severe outcomes in COVID-19.

## 1. Introduction

The COVID-19 pandemic, caused by the severe acute respiratory syndrome coronavirus 2 (SARS-CoV-2), has been a global challenge since its emergence [1]. In Brazil, the number of confirmed cases surpassed thirty-eight million, making it the fifth most affected country in the world. Furthermore, with over 712,000 registered deaths, Brazil holds the second position in mortality rate, according to the Health Ministry (https://covid.saude.gov.br/, 19 September 2024). COVID-19 presents a wide range of clinical manifestations, from asymptomatic to lethal cases [2]. In the acute phase of the disease, patients can progress from mild symptoms, such as cough, fatigue, fever, and sore throat [2,3], to moderate or severe symptoms, which mainly affect the upper respiratory tract, dyspnea, hypoxemia [2,3], and acute respiratory distress syndrome (ARDS) [4]. ARDS, particularly in the context of COVID-19, is closely associated with the development of cytokine storm syndrome (CSS), characterized by an excessive production of pro-inflammatory cytokines, which can lead to severe outcomes, such as ARDS, multiple organ failure, and death) [5,6,7,8]. Elevated inflammatory cytokines in CSS include interleukin (IL)-1β, IL-6, and tumor necrosis factor-alpha (TNF-α) [9]. They have been associated with the progression and severe outcomes of COVID-19 [9,10,11]. Studies have indicated a strong association between elevated levels of IL-1β and severe outcomes in COVID-19, including the development of pulmonary fibrosis and the need for invasive mechanical ventilation [12,13,14,15].

Additionally, high levels of IL-6, as observed in various COVID-19 studies, correlate with predictive mortality and association with respiratory failure, shock, and severe symptoms in hospitalized patients [16,17,18]. Similarly, elevated TNF-α negatively impacts the lower respiratory system, resulting in airway narrowing, bronchial hyperreactivity, damage to the respiratory epithelium, and pulmonary fibrosis [19,20,21]. Several studies reviewed by Qudus et al. (2023) [22] have associated genetic variants with cytokine expression and the development of ARDS through cytokine storm mechanisms. Genetic variants in the promoter region of the *IL1B* gene have been associated with negative outcomes in inflammatory conditions, such as rs4848306 in reactive septic arthritis and rs16944 in chronic obstructive pulmonary disease (COPD). A study showed that the T allele of *IL1B* +3953C > T provides protection against severe COVID-19, while the CT genotype increases the risk of severity [23]. For the *IL6* gene, intronic variants have been related to respiratory diseases, including ARDS and COPD, with the GG genotype of rs1800795 associated with severe COVID-19 cases among Kurdish patients [24]. Variants in the promoter region of the *TNF* gene are associated with a range of clinical conditions and severe outcomes. Additionally, the A allele of the rs1800629 variant has been correlated with increased severity and higher mortality risk associated with COVID-19, suggesting its potential as a genetic marker for predicting adverse outcomes [25]. Other *TNF* variants such as rs361525G, rs1799724, and rs1800630A have also been associated with inflammatory syndromes, like Thrombocytopenia Syndrome (SFTS) and ARDS.

These findings illustrate how genetic variants, particularly in regulatory regions of cytokine genes, may influence host susceptibility to severe disease outcomes. The immune response to SARS-CoV-2 is shaped by a combination of environmental and genetic factors, and the variability in COVID-19 severity—from mild symptoms to fatal complications—highlights the importance of exploring genetic contributions. Despite growing evidence, data on the role of specific immunogenetic variants remain limited, especially regarding their impact on genetically diverse populations. Understanding these associations is essential to clarifying individual variability in immune response and disease progression.

The thirteen SNPs selected in this study are located in promoter or enhancer regions of the *IL1B*, *IL6*, and *TNF* genes and were chosen based on evidence from the HaploReg [26] and ENCODE databases [27], indicating functional regulatory activity. These variants have been associated with transcriptional modulation, altered binding motifs, and epigenetic marks—e.g., histone modifications— in immune-relevant tissues, supporting their role in inflammatory and respiratory responses. Although self-reported ancestry data were not available, the study population represents a genetically admixed cohort from southern Brazil, predominantly of European, African, and Native American backgrounds. To date, few studies have evaluated the association of functionally relevant variants in cytokine genes with COVID-19 severity in Brazilian populations—particularly in the South, where genetic profiles and epidemiological patterns differ from other regions [28]. This study aims to address this gap by investigating the association between thirteen functional variants in *IL1B*, *IL6*, and *TNF* and severe COVID-19 outcomes in 500 unvaccinated individuals from southern Brazil.

## 2. Materials and Methods

### 2.1. Study Design and Data Collection Methodology

The sample consisted of 500 patients who were diagnosed with COVID-19 through the real-time reverse transcription polymerase chain reaction (RT-PCR) test for the detection of SARS-CoV-2 viral RNA, using oro-nasopharyngeal swabs. These samples were collected from April 2020 to March 2021. All the samples were provided by the Central Laboratory of Rio Grande do Sul (LACEN-RS), one of the main laboratories responsible for COVID-19 testing during the pandemic in Rio Grande do Sul, the most southern state of Brazil. Demographic and clinical data were collected from the Brazilian notification system SIVEP-GRIPE (Influenza Epidemiological Surveillance Information System—Severe Acute Respiratory Syndrome Notifications). The clinical and demographic information collected comprised gender, age, permanent place of residence, and comorbidities (including heart diseases, lung diseases, obesity, hematological diseases, Down Syndrome, liver diseases, asthma, diabetes mellitus, immunodeficiencies, kidney diseases, or neurological diseases). Regarding COVID-19, the date of symptom onset, date of the RT-PCR test, date of hospital admission, and symptoms at the time of COVID-19 diagnosis, such as fever, cough, sore throat, difficulty breathing, oxygen saturation rate < 95%, fatigue, and loss of smell or taste, were collected from the form of COVID-19 notification. For COVID-19 cases that required hospitalization, data related to oxygen therapy, intensive care unit care, and outcome (death or recovery) were also recorded from medical records. Following processing and filtering of clinical data, participants were classified into two main groups: hospitalized and non-hospitalized. Additionally, patients were further categorized based on their clinical condition into the following groups: (1) requiring intensive care unit (ICU) admission versus not requiring ICU admission and (2) deceased versus discharged from the hospital. Given that participants were classified according to disease outcomes, this study follows a retrospective case–control design. Clinical outcomes were classified using standardized criteria based on national COVID-19 guidelines [29]. Clinical severity levels were determined using a hierarchical logic as follows:

- 0 (Asymptomatic): Case explicitly recorded as “asymptomatic” in the system;
- 1 (Mild): No ICU admission, no ventilatory support, and no death;
- 2 (Moderate): Received ventilatory support (non-invasive or invasive), but no ICU admission or death;
- 3 (Severe): ICU admission and/or death.

For statistical analyses, individuals classified as moderate (2) and severe (3) were grouped as hospitalized patients. Apart from a confirmed COVID-19 diagnosis, no further exclusion criteria were applied. After curating the clinical data, cases with incomplete or inconclusive information were removed from the final analysis.

### 2.2. DNA Extraction and Genotyping Methods

Genomic DNA was extracted from nasopharyngeal swab samples using the DNA extraction methodology developed by Sbruzzi et al., 2024 [30]. Subsequently, the samples were genotyped for 13 functional variants in the *TNF*, *IL1B*, and *IL6* genes (Table 1), which were selected based on their known roles in immune response modulation and associations with inflammatory diseases.

Genotyping was performed using TaqMan^®^ assays (Thermo Fisher Scientific, Waltham, MA, USA). Real-time PCR amplification was carried out on the StepOnePlus™ Real-Time PCR System, following standard protocols. Quality control criteria included call rates > 98% for all SNPs and >99% concordance in duplicate samples. All genotyped SNPs were evaluated for minor allele frequency (MAF) and tested for deviation from the Hardy–Weinberg equilibrium (HWE). SNPs showing significant deviation from the HWE were further investigated by stratified testing in cases and controls. In addition, all discrepant genotypes were re-genotyped using validated internal controls to confirm the accuracy of the calls. Only SNPs with MAF > 0.01 and appropriate genotyping quality were retained for downstream analysis.

### 2.3. Genetic Association Analysis

To investigate the relationship between cytokine gene variants and COVID-19 severity—including hospitalization, ICU admission, and death—both univariate and multivariate analyses were conducted. Allele and genotype frequencies were compared using Pearson’s chi-square test or Fisher’s exact test. Three genetic inheritance models—additive, dominant, and recessive—were tested for each SNP. Univariate *p*-values were adjusted using the Bonferroni correction to control for multiple testing across the 13 SNPs. Variants with Bonferroni-adjusted *p*-values ≤ 0.100 were retained for multivariate analysis. Multivariate logistic regression models were performed using the Generalized Linear Models (GENLIN) procedure in IBM SPSS Statistics v.18.0.0 (IBM, Armonk, NY, USA; www.ibm.com/products/spss-statistics), specifying a binomial distribution with a logit link function. All models were adjusted for age and sex. No formal matching was performed based on comorbidities. Model significance was evaluated using the likelihood ratio test, and the results were reported as adjusted odds ratios (ORs) with 95% confidence intervals (CIs). A two-tailed *p*-value < 0.05 was considered statistically significant. Missing genotype or covariate data were handled by listwise deletion, implemented via the SPSS command/MISSING CLASSMISSING = EXCLUDE within the GENLIN procedure, ensuring that cases with missing categorical data were excluded from the analysis.

## 3. Results

### 3.1. Symptoms and Comorbidities Associations

The demographic and clinical characteristics of the study sample, stratified by hospitalization status, are presented in Table 2.

Samples with DNA concentrations below 1.5 ng/µL and incomplete clinical data were excluded. The final study included 231 individuals who required hospitalization (46.2%) and 235 subjects who did not require hospital admission (47.0%) (Table 2). Non-hospitalized individuals displayed a significantly lower median age of 38 years (IQR 29.0; 53.0) compared to the hospitalized group (median 65 years; IQR 51.0; 77.0; (*p* < 0.01). There was a higher proportion of females (56.5%) in the non-hospitalized group, while males were more prevalent (56.4%) among the hospitalized cases (*p* = 0.006). Higher prevalence rates of comorbidities were observed among hospitalized individuals, including obesity (81.8%), heart diseases (81.3%), diabetes mellitus (80.5%), and chronic lung diseases (79.3%). These findings showed a strong association between these comorbidities and the need for hospitalization. Regarding the severity indicators, statistical analysis of the hospitalized cases revealed significant *p*-values (<0.01), further supporting the findings. These indicators include the need for hospitalization, oxygen therapy, intensive care unit (ICU) admission, and the outcome (death). Specifically, 51.8% (*n* = 108) of patients were admitted to critical care, 93.8% (*n* = 213) required ventilatory support, and 54.6% (*n* = 125) experienced mortality (Table 2).

### 3.2. Significant Associations Between IL1B and TNF Genetic Variants and COVID-19 Severity

Among the thirteen genotyped variants, six SNPs in the *IL1B* and *TNF* genes demonstrated the strongest and most consistent associations with COVID-19 severity, particularly in relation to hospitalization, ICU admission, and mortality. We evaluated the HWE and MAF for all 13 genotyped SNPs. The variants *IL1B* rs4848306 and *TNF* rs1800630 showed significant HWE deviations in the control group (*IL1B* rs4848306: *p* ≈ 0.04; *TNF* rs1800630: *p* < 0.001). Re-genotyping and comparison with internal samples and population-based data from the ABraOM database confirmed the consistency of the genotyping results. Thus, we maintain both of them in further analyses.

Analysis of genotypic distribution, including dominant and recessive models, was performed and shown in Supplementary Table S1. The results indicated that the AG genotype of the *IL1B* rs16944 variant was significantly associated with hospitalization, with an odds ratio (OR) of 1.98 (95% CI: 1.22–3.23, *p* = 0.006) (Table 3).

Similarly, the comparison between the AA + AG genotypes and GG also demonstrated an association with hospitalization, with an OR of 1.62 (95% CI: 1.04–2.55, *p* = 0.036). For the *IL1B* rs1143627 variant, the CT genotype was associated with hospitalization compared to the CC genotype, with an OR of 1.82 (95% CI: 1.02–3.27, *p* = 0.043). In the case of *TNF* gene variants, the CT genotype for rs1799964 showed a significant association with hospitalization (OR = 1.97; 95% CI: 1.22–3.22, *p* = 0.006). The comparison between the CC + CT and TT genotypes also revealed a significant association, with an OR of 1.93 (95% CI: 1.23–3.06, *p* = 0.005). For rs1800630, the AA genotype was significantly associated with hospitalization, with an OR of 2.37 (95% CI: 1.08–5.33, *p* = 0.034). The comparison between the AA + AC and CC genotypes was also significant, with an OR of 1.76 (95% CI: 1.09–2.84, *p* = 0.021) (Table 3).

When analyzing susceptibility to severity associated with genetic variants, the genes *IL1B*, *IL6*, and *TNF* exhibited statistically significant allelic and genotypic distributions (see Supplementary Tables S2 and S3). Regarding the need for ICU admission, the results revealed that for the *IL1B* rs16944 variant, individuals with the AG genotype presented an 83.4% higher risk of ICU admission compared to those with the GG genotype (OR = 1.83; 95% CI = 1.09–3.10; *p* = 0.022) (Table 4).

Additionally, for the *IL1B* rs1143627 variant, individuals with the CT genotype have a 108.2% higher risk of ICU admission compared to those with the CC genotype (OR = 2.08; 95% CI = 1.12–3.85; *p* = 0.019). Regarding mortality, the CC homozygote for rs1799964 presented an OR of 3.73 (95% CI: 1.21–14.37), indicating that individuals with this genotype have a significantly higher likelihood of death compared to those with the TT genotype (*p* = 0.034) (Table 4). Furthermore, the comparison between the CC + CT and TT genotypes yielded an OR of 1.66 (95% CI: 1.01–2.77), reinforcing the association of the C allele with an increased risk of death relative to the T allele (*p* = 0.049) (Table 5).

Taken together, the results indicate that four functional variants—*IL1B* rs16944 and rs1143627, and *TNF* rs1799964 and rs1800630—were consistently associated with worse clinical outcomes across different genetic models. These associations were observed for hospitalization, ICU admission, and mortality, reinforcing the role of host genetic variation in inflammatory mediators as a potential determinant of COVID-19 severity.

## 4. Discussion

The primary objective of this study was to investigate the correlation between genetic variations in *IL1B*, *IL6*, *TNF*, and critical outcomes of COVID-19. The heterozygous genotype of the rs16944 (*IL1B*) variant and the presence of the A allele showed a significant association with increased susceptibility to severe COVID-19 outcomes, including hospitalization and ICU admission, suggesting a possible contributory role in disease severity. This finding is consistent with the data reported by Balzanelli et al. (2022) [31], who identified the AG genotype as a potential risk factor for COVID-19. The A allele has also been linked to other severe inflammatory conditions, such as chronic obstructive pulmonary disease (COPD) and septic shock, suggesting that it may predispose individuals to exaggerated inflammatory responses. These immune reactions, particularly cytokine storms, are also observed in severe COVID-19 cases and may help contextualize a potential role for *IL1B* variants in modulating disease severity. Specifically, the A allele is known to regulate IL-1β expression, which leads to the overproduction of this pro-inflammatory cytokine in various conditions, including lung cancer [32]. Moreover, the A allele’s association with increased COPD risk in East Asian populations [33] and a higher likelihood of septic shock and death in patients with systemic inflammatory response syndrome (SIRS) [34] raises the possibility that it may promote a stronger inflammatory response, potentially contributing to more severe COVID-19 progression. The rs1143627 variant, in its heterozygous genotype, was significantly associated with an increased risk of hospitalization and ICU admission. This variant has been implicated in various clinical outcomes related to inflammatory and infectious diseases. The C allele is considered a potential independent risk factor for ICU admission in COVID-19 cases [35], and the CC genotype is associated with an increased risk of severe COVID-19 [36]. A key point is that the presence of the C allele disrupts the TATA box in the promoter region of the gene, reducing *IL1B* expression [32,37], as corroborated by studies showing a lower expression rate of this gene [38]. In contrast, the T allele is associated with increased IL-1β expression, which heightens the risk of tuberculosis [39].

The heterozygous genotype of rs1799964 (*TNF*) was significantly associated with COVID-19 hospitalizations, while individuals with the CC genotype had an increased risk of death. Compared to TT, the CC and CT genotypes were associated with higher odds of hospitalization and mortality. The rs1799964 T > C variant of the *TNF* gene has been associated with various clinical outcomes in different inflammatory and infectious conditions. The increased *TNF* expression observed in the CC genotype [40] may exacerbate the inflammatory response, worsening COVID-19. In studies on SARS-CoV, Wang et al. (2008) [41] reported that the CC genotype was associated with severe complications, while the CT genotype was identified as a potential protective factor against SARS-CoV infection. However, Li et al. (2022) [42] found an association between the C allele and a mild COVID-19 phenotype, suggesting that gene expression may play a complex role, with significant variations among different coronavirus infections. The rs1800630 C > A variant was significantly associated with a higher susceptibility to severe COVID-19 outcomes, such as hospitalization, with the AA homozygous showing an elevated risk and AA + AC compared to CC also suggesting a potential genetic predisposition to hospitalization. The A allele has been consistently linked to increased TNF-α expression, as reviewed by Smith and Humphries (2009) [43] and Bank et al. (2014) [37]. Additionally, the A allele was associated with a higher promoter affinity for NF-κB, which may influence gene expression [44]. Clinically, the A allele is related to an increased risk of acute respiratory distress syndrome (ARDS) [45]. In contrast, the CC genotype has been associated with severe outcomes in patients with Severe Fever with Thrombocytopenia Syndrome (SFTS) [46]. Non-hospitalized individuals were notably younger compared to hospitalized ones. This may be owing to the fact that older individuals are more susceptible to both infection and severe COVID-19 cases due to a higher likelihood of having multiple comorbidities [47], in addition to the already observed increase in exhausted T-cell response and a decrease in naive T-helper lymphocytes, as well as a reduction in pro-inflammatory cytokines associated with age [48]. Male individuals were the majority in the hospitalized group, as male sex tends to be associated with greater severity and mortality from COVID-19, while young women exhibited an overrepresentation of pathways related to T- and B-cell activation. This suggests that women may have a more robust immune activation profile. Additionally, males tend to exhibit higher levels of inflammatory cytokines and a lower percentage of lymphocytes during SARS-CoV-2 infection and recovery [49]. The most common comorbidities found in this sample are those already been identified as associated with severe COVID-19 cases, including hypertension, diabetes, cardiovascular diseases, obesity, and, less commonly, respiratory diseases, liver diseases, kidney diseases, and neoplasms [47,50,51,52,53]. In this context, the association between genetic variants in the *IL1B*, *IL6*, and *TNF* genes and the severity of COVID-19 was explored, with particular emphasis on outcomes such as hospitalization, the need for ICU admission, and mortality.

This study has limitations that should be considered. The relatively small sample size may have limited the statistical power to detect modest associations, and the lack of longitudinal follow-up precludes assessment of late clinical outcomes such as reinfections or post-acute complications. These factors may limit the generalizability of the findings. Furthermore, serum cytokine levels were not measured, hindering direct biochemical genotype–phenotype correlation. Although prior experimental studies support the functional relevance of the investigated variants [32,37,38,39,40,41,42,43,44], future research should integrate genetic analysis with cytokine profiling (e.g., ELISA or bead-based multiplex assays) to elucidate the mechanistic link between host genetics, immune modulation, and disease severity. While the lack of cytokine profiling is a limitation in this study, it is important to consider that genotype–phenotype correlations may be observed only in particular situations. Functional variants in genes such as IL6, IL1B, or TNF may only exert measurable effects when their downstream pathways are actively engaged—for instance, during an inflammatory response [42,43,44,45,46,47,48,49,50,51,52,53]. In individuals with low baseline cytokine levels, the same risk alleles may not manifest any detectable phenotypic impact. The context-dependent nature of genotype effects means that they are not fixed but continue to be modulated as a result of immunological state or environmental factors [54]. In the case of COVID-19, in which dysregulated cytokine release is a hallmark of severe disease, genetic variants that promote pro-inflammatory signaling might intensify this response, contributing to worse outcomes [54]. Without protein-level data at the moment of the infection, however, it is not possible to determine whether the observed genetic associations reflect true functional consequences or are conditional upon unmeasured immune activation. Future studies should incorporate cytokine quantification to disentangle constitutive versus context-specific genetic effects. Additionally, common genetic variants, such as those we analyzed in this study, could have a small effect size, and this could make it difficult to identify this association [53].

Data on self-reported ancestry and genomic population structure were not available. This study was conducted in a cohort from southern Brazil, characterized by a genetically admixed background [28]. Population stratification and genetic ancestry are important considerations in genomic studies since differences in allele frequencies across ancestral groups can confound associations if not properly accounted for [55]. Including individuals from diverse ancestral backgrounds increases the chances of identifying new associations and can reveal population-specific effects and motivate future replication efforts in those groups [55,56]. Importantly, this case–control study did not adjust for population stratification, which is critical in admixed populations, such as those in Latin America. In such settings, linkage disequilibrium (LD) patterns vary as a function of ancestral contributions: populations with higher Native American ancestry exhibit slower LD decay, whereas African-derived populations show more rapid decay [57]. This heterogeneity, shaped by historical admixture (e.g., the trans-Atlantic slave trade and post-colonial migrations), influences both haplotype structure and allele frequency distributions, potentially leading to spurious associations in the absence of appropriate correction. Future studies should incorporate ancestry-informative markers or genome-wide principal components to account for population substructure and increase the robustness and replicability of genetic findings.

Additionally, many candidate gene associations—especially those identified in small, single-center cohorts—have failed replication in broader or more diverse populations [58,59]. This often happens due to several factors, including differences in LD structure, environmental exposures, phenotype definitions, or analytical approaches across studies [60,61]. Brazil is a high admixture country [28], and LD structure and allelic frequencies, as well as gene–environment interactions, might make different such associations. Failure to replicate the results does not necessarily negate a true biological signal but may reflect context-dependent effects or unmeasured confounding. This is particularly relevant in populations such as Brazil, where unique population-specific genetic variants—such as loss-of-function variants identified by the DNA do Brasil initiative—may act as unmeasured confounders influencing disease susceptibility and outcomes [28]. Therefore, validation in larger, ancestrally diverse cohorts with harmonized protocols is essential to better understand genetic associations. Key strengths of this study include the cohort of 500 unvaccinated individuals from the pre-vaccine era, the use of national epidemiological registry data (SIVEP-Gripe), and the focus on functional variants in central inflammatory pathways. Multivariate models adjusted for major confounders further strengthen the associations found.

## 5. Conclusions

This study identified significant associations between genetic variants in the *IL1B*, *IL6*, and *TNF* genes and the severity of clinical outcomes in COVID-19 patients. Specific genotypes, such as the AG of rs16944 in the *IL1B* gene and the CT of rs1799964 in the *TNF* gene, were associated with an increased risk of hospitalization, ICU admission, and mortality. These findings suggest that these genetic variants may help to develop strategies of future markers for identifying individuals at higher risk of developing severe forms of the disease. Beyond their relevance to COVID-19, these genetic variants have implications for other viral infections, such as SARS-CoV and severe acute respiratory syndrome, with which they have been associated with severe inflammatory outcomes. The presence of these variants might also influence the immune response to vaccines, affecting both the efficacy and the durability of the protection conferred. As a case–control study conducted in an admixed population with a limited sample size, our findings should be interpreted as hypothesis generating. Replication in larger and more genetically diverse cohorts is required to confirm these associations and to deepen the understanding of their biological implications. In addition, future studies should include extended follow-up periods to better assess the progression of conditions related to genetic variants and the impact of vaccination. Additionally, further studies should explore the interaction of these variants with other genetic and environmental factors to provide a more comprehensive understanding of the genetic influence on the severity of viral infections and vaccine efficacy.

## Figures and Tables

**Table 1:** Variants of the *IL1B*, *IL6*, and *TNF* genes.

Gene	rsID	Positions *	Chr	Type	Probe	Allele	MAF	DNA Do Brasil ** /ABraOM	HaploReg and ENCODE
*IL1B*	rs4848306	−3737	2	Intergenic	C__11725735_10	G/A	A (0.36)	0.36 /0.41	Involved in inflammatory response; proximal enhancer; maximum activity near the *IL1B* gene.
	rs1143623	−1473	2	Intergenic	C___1839941_10	C/G	C (0.29)	0.28 /0.25	Proximal enhancer; involved in the regulation of *IL1B* expression.
	rs16944	−511	2	Intergenic	C___1839943_10	G/A	G (0.49)	0.47 /0.41	Potential inflammatory regulation; located near the *IL1B* gene, with potential regulatory impact.
	rs1143627	−31	2	Intronic	C___1839944_10	C/T	T (0.47)	0.49 /0.43	Potential regulator of inflammatory expression; promoter; maximum activity near *IL1B* gene.
	rs1800795	−174	7	Intronic	C___1839697_20	G/C	C (0.14)	0.21 /0.25	Inflammatory response; promoter; involved in the regulation of *IL6* expression.
*IL6*	rs2069832	+615	7	Intronic	C__15957646_10	G/A	A (0.14)	0.21 /0.25	Inflammation modulation; promoter; related to increased expression of *IL6*.
	rs2069840	+3437	7	Intronic	C__15804104_10	C/G	G (0.19)	0.30 /0.29	Involvement in inflammatory response; intronic; associated with increased expression of *IL6*.
	rs2069845	+3331	7	Intronic	C___1839699_10	G/A	G (0.25)	0.34 /0.34	Involvement in inflammatory response; distal enhancer; related to *IL6* expression.
*TNF*	rs1799964	−1031	6	Intronic	C___7514871_10	T/C	C (0.22)	0.16 /0.25	Potential regulator of inflammatory diseases; proximal enhancer; involved in regulation of *TNF* expression.
	rs1800630	−863	6	Intronic	Customized	C/A	A (0.15)	0.12 /0.18	Regulation of immune response; proximal enhancer; highly relevant for the regulation of *TNF* expression.
	rs1799724	−857	6	Intronic	C__11918223_10	C/T	T (0.10)	0.07 /.011	Essential for immune response; enhancer; involved in the regulation of *TNF*.
	rs1800629	−308	6	Intronic	C___7514879_10	G/A	A (0.09)	0.07 /0.11	Regulation of inflammatory response; intronic; involved in the modulation of *TNF* expression.
	rs361525	−238	6	Intronic	C___2215707_10	G/A	A (0.06)	0.04 /0.06	Promoter; involved in the regulation of *TNF*; essential for immune response.

Chr = chromosome; MAF = minor allele frequency; ** DNA do Brasil Project Variant Browser/ABraOM: Brazilian genomic variants (MAF). * Nucleotide positions are relative to the first ATG codon at exon 1, where the adenine is assigned position +1.

**Table 2:** Demographic and clinical characteristics of COVID-19 patients (hospitalization, ICU, and death) [ *n* (%)].

	Non-Hospitalized (*n* = 235)	Hospitalized (*n* = 231)	*p*	Non-ICU (*n* = 342)	ICU (*n* = 108)	*p*	Discharged from the Hospital (*n* = 337)	Death (*n* = 125)	*p*
Age									
*n*	235	231		342	108		337	125	
Years	38 [29.0; 53.0]	65 [51.0; 77.0]		47 [32.0; 65.0]	64.5 [50.50; 76.75]		44 [31.0; 60.0]	72 [54.0; 80.50]	
Total	52 [36.0; 69.0]		<0.01 *	51.50 (36.75; 63.50)		<0.01 *	52.50 [29.0; 61.75]		<0.01 *
Sex									
Female	139 (56.5)	107 (43.5)		186 (78.2)	52 (21.8)		188 (77.0)	56 (23.0)	
Male	96 (43.6)	124 (56.4)		156 (73.6)	56 (26.4)		149 (68.3)	69 (31.7)	
Total	235 (50.4)	231 (49.6)	0.01 ^‡^	342 (76.0)	108 (24.0)	0.26 ^‡^	337 (72.9)	125 (27.1)	0.04 ^‡^
Comorbidities									
Heart conditions									
No	158 (57.0)	119 (43.0)		214 (79.3)	56 (20.7)		218 (79.3)	57 (20.7)	
Yes	24 (18.7)	104 (81.3)		79 (62.7)	47 (37.3)		67 (52.3)	61 (47.7)	
Total	182 (44.9)	231 (55.1)	<0.01 ^‡^	293 (74.0)	103 (26.0)	<0.01 ^‡^	285 (70.7)	118 (29.3)	<0.01 ^‡^
Chronic lung diseases									
No	175 (47.3)	195 (52.7)		270 (74.8)	91 (25.2)		266 (72.3)	102 (27.7)	
Yes	6 (20.7)	23 (79.3)		19 (65.5)	10 (34.5)		15 (51.7)	14 (48.3)	
Total	181 (45.4)	218 (54.6)	0.01 ^‡^	289 (74.1)	101 (25.9)	0.27 ^‡^	281 (70.8)	116 (29.2)	0.02 ^‡^
Obesity									
No	173 (49.3)	178 (50.7)		266 (77.6)	77 (22.4)		255 (73.1)	94 (26.9)	
Yes	8 (18.2)	36 (81.8)		19 (44.2)	24 (55.8)		23 (52.3)	21 (47.7)	
Total	181 (45.8)	214 (54.2)	<0.01 ^‡^	285 (73.8)	101 (26.2)	<0.01 ^‡^	278 (70.7)	115 (29.3)	0.004 ^‡^
Diabetes mellitus									
No	168 (51.4)	159 (48.6)		252 (78.5)	69 (21.5)		248 (76.1)	78 (23.9)	
Yes	15 (19.5)	62 (80.5)		39 (53.4)	34 (46.6)		36 (47.4)	40 (52.6)	
Total	183 (45.3)	221 (54.7)	<0.01 ^‡^	291 (73.9)	103 (26.1)	<0.01 ^‡^	284 (70.6)	118 (29.4)	<0.01 ^‡^
Severity ^§^									
Admission to critical care (*n* = 414)									
No		116 (51.8)							
Yes		108 (48.2)							
Total		224 (49.8)	<0.01 ^‡^						
Ventilatory support required (*n* = 453)									
No		14 (6.2)							
Yes		213 (93.8)							
Total		227 (50.1)	<0.01 ^‡^						
Death (*n* = 427)									
No		104 (45.4)							
Yes		125 (54.6)							
Total		229 (49.6)	<0.01 ^‡^						

[IQR−–IQR+]; * Mann–Whitney test; ^‡^ chi-square test. ^§^ The present analysis was restricted to hospitalized individuals.

**Table 3:** Multivariate logistic regression analysis for variants of *IL1B*, *IL6*, and *TNF* genes—hospitalization *.

Gene	Variant	Genotype	Odds Ratio (OR)	95% CI	*p*
*IL1B*	rs16944	AA	1.015	0.548–1.879	0.961
		AG	1.976	1.218–3.234	**0.006**
		AA + AG vs. GG	1.620	1.035–2.552	**0.036**
		GG + AG vs. AA	0.697	0.400–1.208	0.200
	rs1143627	TT	0.958	0.526–1.745	0.887
		CT	1.820	1.021–3.269	**0.043**
		TT + CT vs. CC	1.363	0.801–2.330	0.255
		CC + CT vs. TT	0.639	0.405–1.001	0.052
*IL6*	rs2069845	GG	1.006	0.489–2.080	0.987
		AG	0.665	0.420–1.050	0.081
		GG + AG vs. AA	0.724	0.468–1.115	0.143
		AA + AG vs. GG	1.229	0.620–2.455	0.555
*TNF*	rs1799964	CC	1.720	0.684–4.422	0.252
		CT	1.970	1.220–3.215	**0.006**
		CC + CT vs. TT	1.927	1.226–3.060	**0.005**
		TT + CT vs. CC	1.364	0.554–3.426	0.501
	rs1800630	AA	2.367	1.079–5.326	**0.034**
		AC	1.584	0.919–2.681	0.101
		AA + AC vs. CC	1.755	1.094–2.842	**0.021**
		CC + AC vs. AA	2.112	0.976–4.687	0.061
	rs1799724	TT	2.852	0.562–21.694	0.242
		CT	1.411	0.823–2.434	0.212
		TT + CT vs. CC	1.491	0.886–2.529	0.134
		CC + CT vs. TT	2.654	0.524–20.149	0.275
	rs1800629	AA	0.350	0.027–8.621	0.433
		AG	1.054	0.606–1.833	0.852
		AA + AG vs. GG	1.020	0.590–1.762	0.944
		GG + AG vs. AA	0.347	0.027–8.530	0.429

* All models were adjusted for age and sex. Bold numbers indicate statistical significance.

**Table 4:** Multivariate logistic regression analysis for variants of *IL1B*, *IL6*, and *TNF* genes—intensive care unit (ICU) *.

Gene	Variant	Genotype	Odds Ratio (OR)	95% CI	*p*
*IL1B*	rs16944	AA	0.835	0.452–1.559	0.567
		AG	1.834	1.092–3.101	**0.022**
		AA + AG vs. GG	1.425	0.891–2.272	0.138
		GG + AG vs. AA	0.608	0.349–1.077	0.083
	rs1143627	TT	1.085	0.587–1.983	0.793
		CT	2.082	1.123–3.848	**0.019**
		TT + CT vs. CC	1.526	0.873–2.626	0.131
		CC + CT vs. TT	0.669	0.418–1.073	0.094
*IL6*	rs2069845	GG	1.590	0.715–3.852	0.276
		AG	0.653	0.400–1.061	0.086
		GG + AG vs. AA	0.776	0.485–1.233	0.285
		AA + AG vs. GG	1.979	0.934–4.630	0.091
*TNF*	rs1799964	CC	1.844	0.687–5.908	0.256
		CT	1.252	0.762–2.087	0.381
		CC + CT vs. TT	1.328	0.827–2.158	0.245
		TT + CT vs. CC	1.707	0.646–5.404	0.315
	rs1800630	AA	1.474	0.651–3.689	0.376
		AC	1.083	0.633–1.893	0.773
		AA + AC vs. CC	1.175	0.722–1.940	0.521
		CC + AC vs. AA	1.442	0.646–3.572	0.396
	rs1799724	TT	…	…	…
		CT	…	…	…
		TT + CT vs. CC	1.218	0.702–2.179	0.493
		CC + CT vs. TT	…	…	…
	rs1800629	AA	0.568	0.025–6.342	0.654
		AG	0.541	0.262–1.037	0.077
		AA + AG vs. GG	0.542	0.269–1.024	0.710
		GG + AG vs. AA	0.621	0.028–6.931	0.706

* All models were adjusted for age and sex. Note **:** “…” indicates that estimates could not be calculated due to an insufficient number of observations. Bold numbers indicate statistical significance.

**Table 5:** Multivariate logistic regression analysis for variants of *IL1B*, *IL6*, and *TNF* genes—death *.

Gene	Variant	Genotype	Odds Ratio (OR)	95% CI	*p*
*IL1B*	rs16944	AA	0.704	0.365–1.362	0.295
		AG	1.274	0.750–2.168	0.370
		AA + AG vs. GG	1.065	0.652–1.731	0.799
		GG + AG vs. AA	0.617	0.342–1.123	0.110
	rs1143627	TT	1.357	0.709–2.590	0.354
		CT	1.615	0.860–3.028	0.134
		TT + CT vs. CC	1.493	0.833–2.653	0.173
		CC + CT vs. TT	0.979	0.601–1.607	0.933
*IL6*	rs2069845	GG	1.266	0.579–2.882	0.563
		AG	0.930	0.562–1.537	0.776
		GG + AG vs. AA	0.990	0.614–1.594	0.966
		AA + AG vs. GG	1.313	0.627–2.877	0.481
*TNF*	rs1799964	CC	3.728	1.208–14.366	**0.034**
		CT	1.452	0.863–2.479	0.165
		CC + CT vs. TT	1.658	1.008–2.766	**0.049**
		TT + CT vs. CC	3.279	1.081–12.472	0.052
	rs1800630	AA	1.498	0.652–3.668	0.355
		AC	1.619	0.904–2.973	0.112
		AA + AC vs. CC	1.583	0.945–2.700	0.086
		CC + AC vs. AA	1.328	0.587–3.205	0.509
	rs1799724	TT	…	…	…
		CT	…	…	…
		TT + CT vs. CC	1.682	0.931–3.141	0.092
		CC + CT vs. TT	…	…	…
	rs1800629	AA	1.386	0.112–33.456	0.805
		AG	1.033	0.550–1.895	0.918
		AA + AG vs. GG	1.047	0.566–1.898	0.880
		GG + AG vs. AA	1.379	0.111–33.228	0.808

* All models were adjusted for age and sex. Note **:** “…” indicates that estimates could not be calculated due to an insufficient number of observations. Bold numbers indicate statistical significance.

## Data Availability

The data that support the findings of this study are available from the corresponding author upon reasonable request. Due to ethical and legal restrictions, the data are not publicly available.

## Supplementary Materials

The following supporting information can be downloaded at https://www.mdpi.com/article/10.3390/diagnostics15111403/s1. Supplementary Table S1—Genotypic frequencies and analysis of dominant and recessive models for variants in the *IL1B*, *IL6*, and *TNF* genes [*n* = (%)]. (Hospitalization.) Supplementary Table S2—Genotypic frequencies and analysis of dominant and recessive models for variants in the *IL1B*, *IL6*, and *TNF* genes [*n* = (%)]. (Intensive care unit—ICU). Supplementary Table S3—Genotypic frequencies and analysis of dominant and recessive models for variants in the *IL1B*, *IL6*, and *TNF* genes [*n* = (%)]. (In relation to death).
